# The Disassociation of A3G-Related HIV-1 cDNA G-to-A Hypermutation to Viral Infectivity

**DOI:** 10.3390/v16050728

**Published:** 2024-05-04

**Authors:** Joanie Martin, Xin Chen, Xiangxu Jia, Qiujia Shao, Bindong Liu

**Affiliations:** 1 Center for AIDS Health Disparities Research, Department of Microbiology, Immunology and Physiology, School of Medicine, Meharry Medical College, Nashville, TN 37208, USA; jmartin19@email.mmc.edu (J.M.); xin.chen@vanderbilt.edu (X.C.); xjia@mmc.edu (X.J.); qshao@mmc.edu (Q.S.); 2 School of Graduate Studies, Meharry Medical College, Nashville, TN 37208, USA

**Keywords:** APOBEC3G, cytidine deaminase, G-to-A hypermutation, HIV-1 Vif, proteasomal degradation, encapsidation, viral cDNA, next-generation sequencing

## Abstract

APOBEC3G (A3G) restricts HIV-1 replication primarily by reducing viral cDNA and inducing G-to-A hypermutations in viral cDNA. HIV-1 encodes virion infectivity factor (Vif) to counteract A3G primarily by excluding A3G viral encapsidation. Even though the Vif-induced exclusion is robust, studies suggest that A3G is still detectable in the virion. The impact of encapsidated A3G in the HIV-1 replication is unclear. Using a highly sensitive next-generation sequencing (NGS)-based G-to-A hypermutation detecting assay, we found that wild-type HIV-1 produced from A3G-expressing T-cells induced higher G-to-A hypermutation frequency in viral cDNA than HIV-1 from non-A3G-expressing T-cells. Interestingly, although the virus produced from A3G-expressing T-cells induced higher hypermutation frequency, there was no significant difference in viral infectivity, revealing a disassociation of cDNA G-to-A hypermutation to viral infectivity. We also measured G-to-A hypermutation in the viral RNA genome. Surprisingly, our data showed that hypermutation frequency in the viral RNA genome was significantly lower than in the integrated DNA, suggesting a mechanism exists to preferentially select intact genomic RNA for viral packing. This study revealed a new insight into the mechanism of HIV-1 counteracting A3G antiviral function and might lay a foundation for new antiviral strategies.

## 1. Introduction

Human A3G belongs to the APOBEC3 (A3) family of evolutionarily conserved cytidine deaminases. A3 enzymes have become notorious for their role in viral immunity as they pertain to intrinsic responses to infections by retroviruses, including HIV-1 and other primate lentiviruses [1,2,3]. Of the A3 family members, A3G, also known as CEM15, possesses the most potent antiviral effects; it is most notably known for deaminating viral cytosine (C) to uracil (U) in the negative strand cDNA replication intermediate, which transfers from guanosine (G) to adenine (A) hypermutation during plus-strand synthesis. It also has effects on the process of reverse transcription and viral DNA integration [1,2,3]. HIV-1 encodes a 23-kDa protein known as viral infectivity factor or Vif, a virulence factor that promotes viral replication and persistence in permissive cell types [4,5]. The predominant function of Vif is to overcome A3G-mediated restriction by inducing proteasomal degradation, which excludes A3G from encapsidating into budding virions [6,7,8,9]. The Vif-mediated degradation process is initiated after Vif simultaneously binds to A3G and a cellular E3 ubiquitin ligase complex consisting of Cullin5, Elongin B/C, RING-box protein 2 (RBX2), and core binding factor (CBF-β) [9,10,11]. This complex induces the polyubiquitination of A3G, which is a marker for proteasomal degradation. There’s also evidence that Vif has the capacity to reduce the encapsidation of A3G through degradation-independent mechanisms, such as impairing A3G translation or intracellular stability [12,13,14]. 

Even though the Vif-induced degradation of A3G is robust and most of A3G is excluded from HIV-1 virions, substantial evidence from laboratory and clinical studies supports the idea that residual amounts of A3G are still encapsidated into the HIV-1 virion. It is well known that the genome of HIV-1 primary isolates possesses G-to-A hypermutations and that the hypermutation preferentially happens to the GG and GA dinucleotides sequence [15,16,17,18,19,20,21,22,23]. Since GG and GA are the hotspots of A3G and A3F mutation sites, the existence of G-to-A hypermutation in the genome of HIV primary isolates suggests that a certain amount of A3G and A3F molecules still exist inside the virion. Indeed, despite the robust potential of Vif to limit the encapsidation of A3G, a detectable amount of A3G is still present in the budding virion. Nowarski et al. demonstrated that purified wild-type virions produced in H9 cells, a CD4^+^ T cell line, contained A3G molecules with deaminase activity [24]. Furthermore, Gillick et al. and Xu et al. showed that detectable amounts of A3G are present in wild-type HIV-1 particles produced from CD4^+^ T and peripheral blood mononuclear cells (PBMCs) during infection [25,26]. 

Two independent studies have shown that only a few A3G molecules (1 or 3-11) can completely inhibit Vif-deficient HIV-1 replication [26,27]. The detailed action (or actions) of encapsidated A3G to wild-type HIV-1 replication and how HIV-1 overcomes this action is yet to be fully elucidated. In this study, we provide evidence that encapsidated A3G from Vif-proficient HIV-1 induces G-to-A hypermutations in newly synthesized viral cDNA, however, fails to inhibit viral replication. Using a highly sensitive, NGS-based G-to-A hypermutation detecting assay, we detected a much lower G-to-A hypermutation frequency in viral gRNA than in the integrated viral DNA. The data implies that HIV-1 evolved an undiscovered mechanism to overcome the A3G antiviral function by selectively encapsidating un-impacted viral RNA into newly released virions to maintain its infectivity. Further investigation of the new mechanism may shed light on revealing the complete picture of HIV-1 and A3G interactions and lay a foundation for new antiviral design. 

## 2. Materials and Methods

### 2.1. Cells, Plasmids, Antibodies, and Reagents 

The following cell lines or reagents were obtained through the NIH HIV Reagent Program, Division of AIDS, NIAID, NIH: H9 cells (ARP-87) and HIV-1 IIIB-infected H9 cells (ARP-400) from Dr. Robert Gallo [28,29,30]; HEK-293T cells from Andrew Rice; HeLa CD4^+^ HIV-1 LTR-β-gal cells (MAGI, ARP-1470) from Dr. Michael Emerman; Sup-T1 cells (ARP-100) from Dr. Dharam Ablashi [31,32]; Polyclonal Anti-human APOBEC3G, C-terminal (anti-rabbit, ARP-10201) from by Dr, Jaisri Lingappal; HIV-1 NL4-3ΔEnv-EGFP Reporter Vector (ARP-11100) from Dr. Haili Zhang, Dr. Yan Zhou and Dr. Robert Siliciano [33]. A3G expression plasmid was a gift from Yong-Hui Zheng at the University of Illinois [34]. VSV-G envelope expression vector pMD2.G was a gift from Didier Trono (Addgene plasmid # 12259; http://n2t.net/addgene:12259 (accessed on 1 March 2024); RRID: Addgene_12259). Human anti-Ribosome P antibody (HPO-0100) was purchased from ImmunoVision. UNG Mouse Monoclonal Antibody (TA503563) was obtained from Ori Gene (Rockville, MD, USA). H9 and Sup-T1 cells were cultured in a regular RPMI medium containing 10% fetal calf serum (FBS). HeLa-MAGI-CCR5 cells were grown in a DMEM medium containing 10% FBS. All cells were cultured in a 5% CO_2_ atmosphere.

### 2.2. A3G, UNG2 CRISPR Cas9 Knockout 

The guide RNA for A3G knockout (5′-CUGGGACCCAGAUUACCAGG-3′) was designed using the guide RNA tool design from Benchling. The design of the guide RNA for UNG2 knockout (5′-CGUCUUCUGGCCGAUCAUCC-3′) was published by Sarno et al. [35]. The guide RNA sequences were submitted to Synthego to synthesize sgRNA Kits for A3G and UNG2 knockout. TrueGuide™ sgRNA Negative Control from ThermoFisher was used as a non-targeting negative control (NTC). The sgRNA was mixed with Synthego SpCas9 2NLS Nuclease (3:1) to form a ribonucleoprotein (RNP) complex. The RNP complex was transfected into either H9 or Sup-T1 cells using the Neon Transfection System (ThermoFisher MKP1025, Waltham, MA, USA). The transfection conditions for H9 cells were voltage: 1500 V; width: 10 ms; pulse: 3 pulses; and for Sup-T1, voltage: 1600 V; width: 20 ms; pulse: 1 pulse. Three days post-transfection, genomic DNA was isolated using the published method [36] and subjected to PCR amplification. The primer sequence for A3G PCR amplification is forward: 5′-CAGACAAACACTCAAACCGAACAGG-3′; and reverse: 5′-TATGTGTGGGAAGGACCCATCA-3′. The primer sequence for UNG2 PCR amplification is forward: 5′-AAGAGCCTGTCCAAAGAGCA-3′; and reverse: 5′-CCCGGGTTTACCGAGTCAG-3′. The PCR product was cleaned using the QIAquick PCR Purification kit (QIAGEN 28104, Germantown, MD, USA) and then subjected to DNA Sanger sequencing at Genewiz using the forward primers. The knockout score was evaluated via Synthego ICE analysis (https://ice.synthego.com/#/, accessed on 1 March 2024), and the protein expression was confirmed using Western blot analysis. The UNG2 knockout in Sup-T1 and H9 cells was subsequently cloned using BD FACSAria III cell sorter. One clone of each was selected, further confirmed, and used for later experiments. The knockout score of the A3G knockout H9 cell line was 99%. It was straightly used for experiments. 

### 2.3. Virus Preparation and Infection

HIV-1 IIIB virus (IIIB/H9) was harvested from HIV-1 IIIB-infected H9 cells. To produce the IIIB/Sup-T1 virus, IIIB/H9 was used to infect the Sup-T1 cells through spinoculation [37]. The infected cells were washed three times using PBS after spinoculation. Three days after infection, the cells were washed once more using PBS. Three days after the last wash, IIIB/Sup-T1 was harvested from the culture. The same method was used for the IIIB/H9 virus to infect H9ΔA3G or H9ΔUNG2 to produce IIIB/H9ΔA3G or IIIB/H9ΔUNG2 viruses, respectively. Meanwhile, IIIB/H9 was used to infect H9 cells to produce IIIB/H9 control virus. HEK 293T cells (3 × 10^6^) were seeded 24 h prior to co-transfection with NL4-3ΔEnv (10 μg) and pMD2.G (10 μg), with or without A3G expression vector (5 μg), to produce NL4-3 and NL4-3/A3G viruses using the PEI transfection method [38]. Six hours post-transfection, the cells were washed three times using PBS. An HIV-1 p24CA Antigen Capture Assay Kit (AIDS and Cancer Virus Program, Leidos Biomedical Research, Inc., Frederick National Laboratory for Cancer Research; Frederick, MD, USA) was used to titrate the viral concentrations. 

### 2.4. NGS-Based G-to-A Hypermutation Detecting Assay

Sup T1 cells (1 × 10^6^) were infected with ~250 ng p24CA content of the indicated virus via spinoculation for 2 h at 1300 g, then placed directly to incubate at 37 °C for an additional 1 h. The cells were washed three times with PBS and then incubated at 37 °C, 5% CO_2_ for the indicated time. After incubation, cells were used for DNA isolation using the DNeasy DNA isolation kit (Qiagen; Germantown, MD, USA). Culture supernatant was harvested for RNA isolation using Bio-Rad Aurum™ Total RNA Mini Kit (Bio-Rad; Hercules, CA, USA). Viral RNA was reverse transcribed to cDNA using the High-Capacity cDNA Reverse Transcription Kit (ThermoFisher; Waltham, MA, USA). A 199 bp DNA fragment, a part of the published region by Konning et al. [39] within the nef and LTR region of the HIV-1 genome, was selected for the G-to-A hypermutation analysis (Figure S1). The primer set Forward 5′-CTRATATCRARCTTRCTACAA-3′ and Reverse 5′-TGAGGYTTAAGYAGTGGGTT-3′ were used to amplify the 199 bp DNA fragment from IIIB viral cDNA. The primers set Forward 5′-CTRACATCRARCTTRCTACAA-3′ and 5′-TGAGGYTTAAGYAGTGGGTT-3′ were used to amplify the 199 bp DNA fragment from NL4-3 cDNA. Nested Alu PCR was used to measure the G-to-A hypermutation frequency of integrated viral DNA. Forward primer 5′-AAGCCAACAAAGGAGAGAACACC-3′ and Alu reverse SB704 primer 5′-TGCTGGGATTACAGGCGTGAG-3′ were used for the first round of Nested Alu PCR. The second round of Nested PCR was performed using primers for IIIB viral cDNA mentioned above. To ensure the product of Nested Alu PCR was integrated with viral DNA specifically without unintegrated viral DNA contamination, we adjusted the input DNA to the right amount so that only the sample with DNA Taq polymerase added a show band in the second round of the Nested PCR. The PCR was performed on a Bio-Rad Thermo Cycler PCR Detection System using the following program: 95 °C (3 min); 23 cycles of 94 °C (30 s), 50 °C (30 s), and 72 °C (4 m); 72 °C (6 m) for the first round of the Alu PCR reaction and 94 °C (30 s); 35 cycles of 94 °C (20 s), 58 °C (30 s), 68 °C (5 m), and 12 °C (hold) for the viral cDNA reaction. The PCR products were purified using a QIAquick PCR Purification kit (QIAGEN 28104), measured using Qubit, and submitted to Genewiz for Amplicon EZ analysis. A Qiagen CLC Genomic Workbench was used to analyze the NGS data and calculate the G-to-A hypermutation frequency.

### 2.5. SDS-PAGE and Western Blot

Cells were lysed using RIPA buffer (10 mM of Tris-Cl [pH 8.0] containing 1 mM of EDTA, 0.5 mM of EGTA, 1% Triton X-100, 0.1% sodium deoxycholate, 0.1% SDS, 140 mM of NaCl, and fresh 1 mM of PMSF). The cells were lysed on ice for 30 m. The supernatant was mixed with 4XSDS-PAGE sample buffer (Bio-Rad, Hercules, CA, USA) and subjected to SDS-PAGE. The resultant protein bands were transferred to the nitrocellulose membrane (Bio-Rad) using the Bio-Rad Trans-Blot Turbo Transfer System. Anti-APOBEC3G, anti-UNG, and anti-Ribosome P were used for probing A3G, UNG, and Ribosome P. Super Signal West Dura substrate was used as the substrate. Bio-Rad ChemiDoc MP was used to visualize the protein bands.

### 2.6. MAGI Assay and qRT-PCR

Virus infectivity was measured according to the protocol provided via the MAGI assay, which was adapted from the TZM-bl infectivity assay. MAGI cells were infected with serial dilutions of the virus sample in a complete medium with 20 µg/mL of DEAE-Dextran in a 37 °C, 5% CO_2_ incubator. After a 3 h incubation period, the culture volume was increased 1-fold using the complete medium. After a 48 h incubation, cells were stained with the ß-Galactosidase staining buffer as described [40]. The input virus for infection was normalized via qRT-PCR as described [41].

### 2.7. Statistical Analysis

A paired *t*-test was performed using GraphPad Prism for all NGS-based G-to-A hypermutation and viral infectivity assay analyses. For analyzing the *p*-value of NGS-based G-to-A hypermutation, the hypermutation frequency of each nucleotide position (G to A) was compared to its control using a paired *t*-test. A *p*-value of ≤0.05 was considered statistically significant (*). A *p*-value of ≤0.01 was considered statistically very significant (**).

## 3. Results

### 3.1. HIV Released from A3G-Expressing Cells Generates G-to-A Hypermutation in Viral cDNA

**Figure 1:**
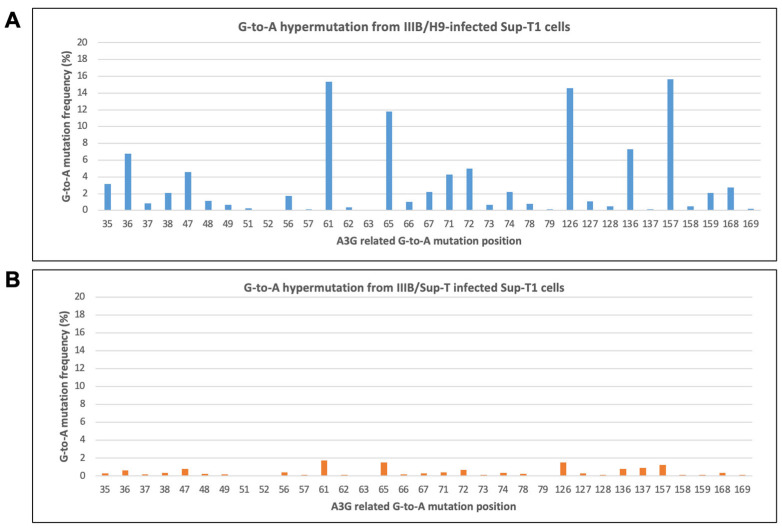

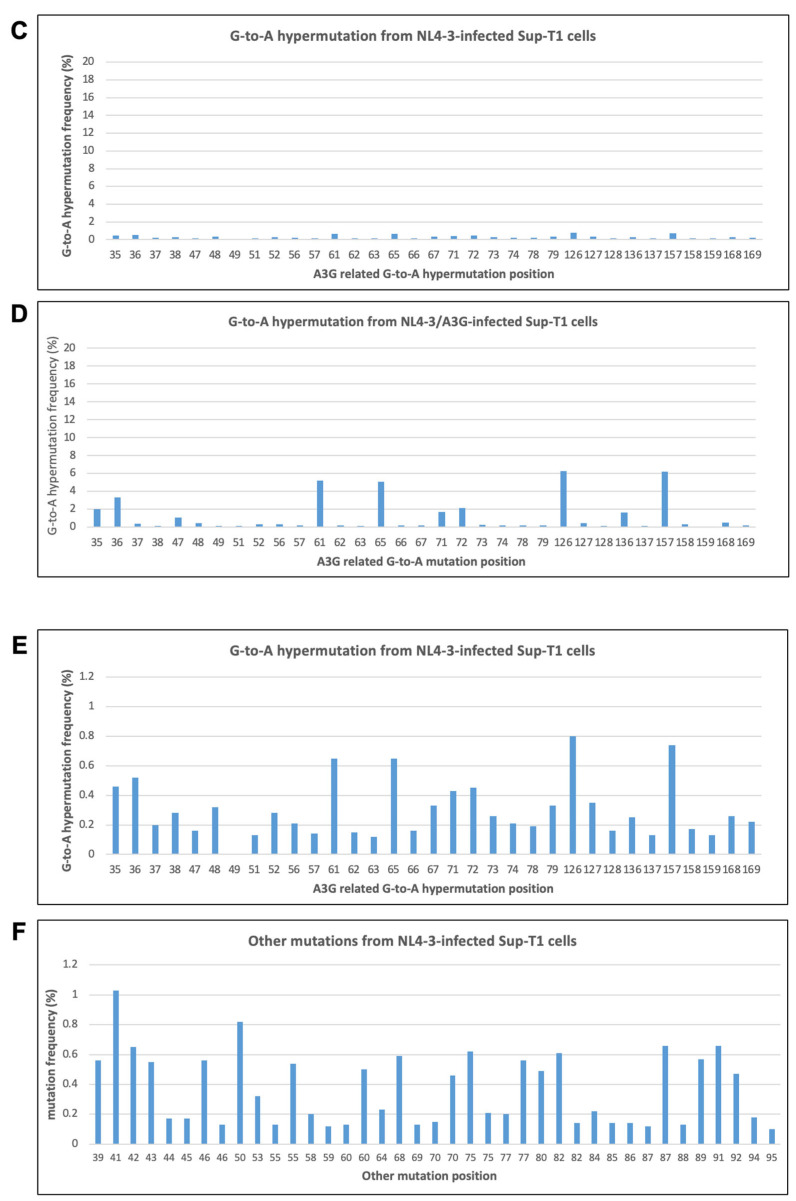
Encapsidated A3G-induced G-to-A hypermutation in viral cDNA. HIV-1 IIIB/H9 (**A**), IIIB/Sup-T1 (**B**), NL4-3 (**C**), and NL4-3/A3G (**D**) were used to infect Sup-T1 cells through spinoculation for 3 h. After three PBS washes, the cells were incubated for 19 h (**A**,**B**) or 6 h (**C**,**D**). After infection, total cellular DNA was isolated, and the PCR product was amplified for NGS-based G-to-A hypermutation detection. The results shown are representative of two independent experiments. (**E**,**F**) contain the same data as (**C**) but shown on a different scale. (**A**–**E**) show A3G-related G-to-A hypermutation. (**F**) shows mutations related to A, T, and C nucleotides.

The literature showed that Vif excludes A3G from virions but does so incompletely, leaving residual A3G in the virion. However, it is unclear whether the residual A3G has any influence on HIV-1 replication. To examine the function of the encapsidated A3G in HIV-1 replication, we first examined whether it could induce G-to-A hypermutation in newly synthesized viral cDNA. To carry this out, we infected Sup-T1 cells using the HIV-1 IIIB virus, harvested from H9 cells chronically infected with HIV-1 IIIB (term it IIIB/H9). Nineteen hours post-infection, total DNA was isolated and subjected to PCR as described in the methods. The PCR product was sent to Genewiz for Amplicon EZ analysis, a streamlined version of NGS to analyze heterogeneous PCR products. As shown in Figure 1A, IIIB/H9 induced G-to-A hypermutation in the viral cDNA of Sup-T1 cells, and greater than 2% of the hypermutation frequencies were found in many positions. It has been shown that HIV-1 released from H9 cells contains A3G in its virion [24]. Therefore, the hypermutation generated by IIIB infection is possibly related to encapsidated A3G. To further confirm this, we infected Sup-T1 cells with the IIIB/H9 virus and passed it on for several generations. It is well known that the Sup-T1 cell does not express A3G. Therefore, after several passages of the IIIB/H9 virus in the Sup-T1 cells, we expect the IIIB virus (term it IIIB/Sup-T1) will not contain A3G in its virion. Indeed, when we repeated the experiment in Figure 1A using IIIB/Sup-T1, there was a lower G-to-A hypermutation frequency that failed to reach above 2% compared to IIIB/H9 (Figure 1B). Together, the data showed that encapsidated A3G induced G-to-A hypermutation in newly synthesized viral cDNA even in the presence of functional Vif.

We also repeated the experiment with an NL4-3 virus, produced from NL4-3ΔEnv proviral construct co-transfected into 293T cells with constructs expressing VSV-G envelope and A3G (NL4-3/A3G) or with VSV-G envelope expression only (NL4-3). Similar results were obtained in Figure 1A,B. NL4-3/A3G induced higher G-to-A hypermutation in viral cDNA. Notice that G-to-A hypermutation was also detected in viral cDNA from cells infected with the NL4-3 virus (without A3G) (Figure 1C). The mutation frequencies were generally lower than 0.6% (Figure 1E). However, when looking at the frequencies of other mutations in the samples (Figure 1F), we also observed up to 0.6% mutations. The data suggest that the low-frequency mutations may come from system errors of the method, spontaneous mutations, and errors from reverse transcription, which might not reflect A3G activity specifically.

### 3.2. Encapsidated A3G Has No Effects on Viral Infectivity

**Figure 2:**
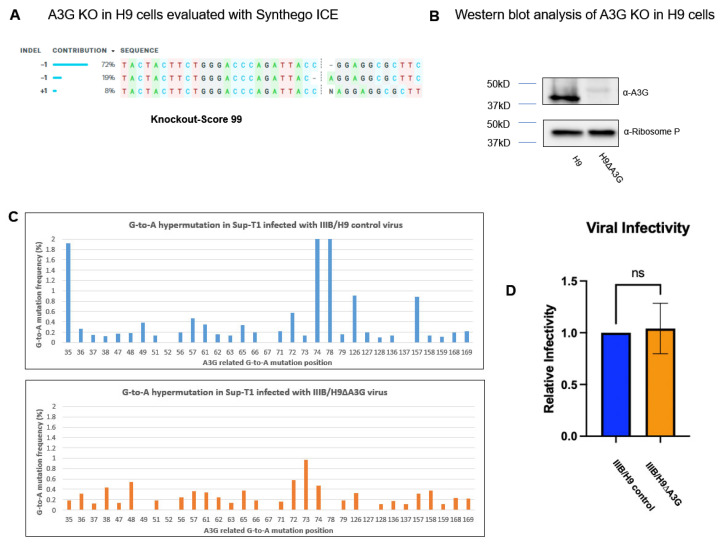
Encapsidated A3G has no effects on viral infectivity. CRISPR Cas9 technology was used to knockout the A3G gene in H9 cells. After 72 h of recovery, total DNA was isolated from the cells, and the A3G gene was amplified via PCR and then subjected to a Sanger sequencing analysis. The A3G knockout score was evaluated with Synthego ICE (**A**). The knockout efficiency of A3G protein expression was analyzed with Western blot (**B**). The IIIB virus harvested from H9 and H9ΔA3G was used to infect Sup-T1 cells. G-to-A hypermutation was analyzed as above (**C**). The results shown are representative of two independent experiments. The infectivity of the IIIB virus harvested from H9 and H9ΔA3G was analyzed via MAGI assay (**D**). The results shown are representative of three independent experiments. A Student’s *t*-test analysis showed no significant difference in infectivity between the two viruses (*p* = 0.7591).

To investigate whether the aforementioned G-to-A hypermutation in cDNA has an impact on the viral infectivity of the released HIV-1, we employed the CRISPR Cas9 system to generate a stable knockout (KO) of A3G from H9 cells as described in the methods. The KO score was determined via the Synthego ICE Analysis with a predicted KO score of 99% (Figure 2A). The efficiency of A3G KO was further verified via Western blot analysis (Figure 2B). H9 and H9ΔA3G cells were subjected to infection with IIIB/H9 to produce IIIB/H9 control and IIIB/H9ΔA3G virions, respectively. Sup-T1 cells were infected for 16 h with the two viruses, and G-to-A hypermutation in viral cDNA was analyzed. The results showed that most G-to-A hypermutation generated via IIIB/H9ΔA3G were below 0.6%, considered non-A3G specific. The IIIB/H9 control virus induced recognizable G-to-A hypermutation (Figure 2C). Next, we measured the infectivity of the IIIB/H9 control and IIIB/H9ΔA3G via the MAGI assay. MAGI cells were infected with the two viruses for 48 h and then stained with a ß-Galactosidase staining kit. As shown in Figure 2D, there was no significant difference in infectivity between either virus even though the IIIB/H9 control induced higher G-to-A hypermutation than IIIB/H9ΔA3G in viral cDNA (*p* = 0.7591). Inducing G-to-A hypermutation has been considered a hallmark of A3G antiviral function. Our data here showed a disconnection between inducing G-to-A hypermutation and A3G antiviral function. Investigating the disconnection will help us better understand the A3G and HIV-1 interaction.

### 3.3. UNG2 Knockout Increases G-to-A Hypermutation Frequency without Decreasing Viral Infectivity

**Figure 3:**
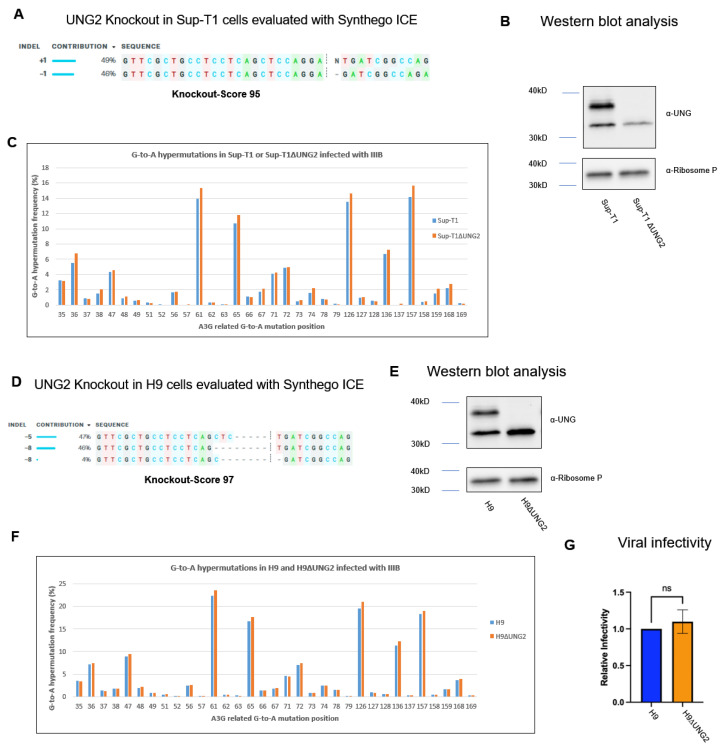
UNG2 knockout increases G-to-A hypermutation frequency without altering viral infectivity. The CRISPR Cas9 method was used to knockout the UNG2 gene in Sup-T1 and H9 cells. Synthego ICE was used to estimate the knockout score for Sup-T1 (**A**) and H9 (**D**). The knockout efficiency of UNG2 protein expression was analyzed via Western blot (**B**,**E**). The IIIB/H9 virus was used to infect Sup-T1 and Sup-T1ΔUNG2, or H9 and H9ΔUNG2 cells, and the G-to-A hypermutation frequency was analyzed (**C**,**F**). A Student’s *t*-test showed significant changes in hypermutation frequency between Sup-T1 and Sup-T1ΔUNG2 cells (*p* = 0.0003) (**C**) and H9 and H9ΔUNG2 cells (*p* = 0.005) (**F**). The infectivity of the IIIB virus harvested from H9 and H9ΔUNG was analyzed via MAGI assay (**G**). A Student’s *t*-test showed no significant difference in infectivity between the two viruses (*p* = 0.1857). The results shown are representative of three independent experiments.

Human uracil DNA glycosylase 2 (UNG2) is an enzyme that plays a crucial role in DNA repair by removing uracil from DNA molecules, which counteracts A3G activity in inducing G-to-A hypermutation. We investigated whether manipulating UNG2 could alter A3G-induced G-to-A hypermutation frequency in viral cDNA and HIV-1 infectivity. To carry this out, we employed the CRISPR Cas9 system to generate a stable KO of UNG2 from Sup-T1 (Sup-T1ΔUNG2) cells as described in the methods. The KO score was determined via Synthego ICE analysis with a predicted KO score of 95% (Figure 3A). The KO efficiency of UNG2 was further verified via Western blot analysis (Figure 3B). To test whether UNG2 KO alters A3G-induced G-to-A hypermutation frequency, the IIIB/H9 virus was used to infect Sup-T1 and Sup-T1ΔUNG2, or H9 and H9ΔUNG2 cells, respectively. The G-to-A hypermutation frequency was analyzed using the Genewiz Amplicon EZ service. As shown in Figure 3C,F, the G-to-A hypermutation frequency was significantly increased in viral cDNA from Sup-T1ΔUNG2 (*p* = 0.0003) and H9ΔUNG2 (*p* = 0.005) cells compared to one from Sup-T1 and H9 cells, respectively. To test whether UNG2 KO alters viral infectivity, we introduced UNG2 KO in H9 cells (Figure 3D,E). The IIIB/H9 virus produced from H9 (IIIB-H9 control) and H9ΔUNG2 (IIIB-H9ΔUNG2) was subjected to a MAGI assay to measure viral infectivity. Although the G-to-A hypermutation significantly increased after UNG2 KO, viral infectivity was unchanged (*p* = 0.3979) (Figure 3G). Once again, as we observed in Figure 2, there was a disconnection between G-to-A hypermutation and viral infectivity.

### 3.4. Viral gRNA G-to-A Hypermutation Rate Is Unchanged despite the Increased G-to-A Hypermutation Frequency in Viral cDNA

**Figure 4:**
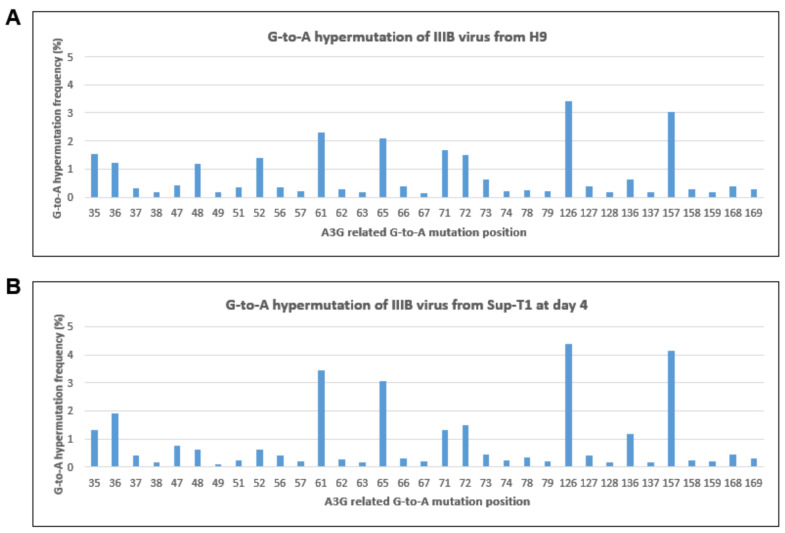
Viral gRNA G-to-A hypermutation frequency is unchanged despite the increased G-to-A hypermutation frequency in viral cDNA. Viral gRNA extracted from the IIIB/H9 stock virus before (**A**) and after a 4-day infection cycle (**B**) in Sup-T1 cells was subjected to RT-PCR. The G-to-A hypermutation frequency of the RT-PCR product was analyzed using the NGS-based G-to-A hypermutation detecting assay (**A**,**B**). A Student’s *t*-test showed no significant difference in G-to-A hypermutation between the two viruses (*p* = 0.1347).

We observed the disconnection between G-to-A hypermutation and viral infectivity from the data in Figure 3 and Figure 4. Next, we decided to analyze the G-to-A hypermutation frequency in viral genomic RNA. The IIIB virus from Sup-T1 cells infected with IIIB/H9 for up to 4 days was subjected to RNA isolation. The RT-PCR product of the gRNA was analyzed using Genewiz Amplicon EZ services. Input IIIB/H9 (from chronically infected H9) was included to compare the hypermutation frequency of the viral genome before infection and after virus release from Sup-T1 cells. The G-to-A hypermutation frequency of viral gRNA from IIIB/H9 input and IIIB/H9 4 days post-infection showed no significant difference (*p* = 0.1347) (Figure 4). Interestingly, the G-to-A hypermutation frequency of IIIB/H9 stock (Figure 4A) and IIIB/H9 day 4 post-infection (Figure 4B) are dramatically lower than the frequency in viral cDNA of 19 h post-infection (Figure 1A). The data indicated that the G-to-A hypermutation of viral gRNA remains at steady low levels even though high levels of G-to-A hypermutation have been observed in viral cDNA during its replication. It answered why there was a disconnection between G-to-A hypermutation in cDNA and viral infection, as shown in Figure 2 and Figure 3, because the G-to-A hypermutation frequency in viral gRNA was kept at steady low levels. The data also suggest that there might be a selection of viral gRNA encapsidation that preferentially selects un-impacted viral gRNA for encapsidation.

### 3.5. G-to-A Hypermutation Frequency in Integrated cDNA

**Figure 5:**
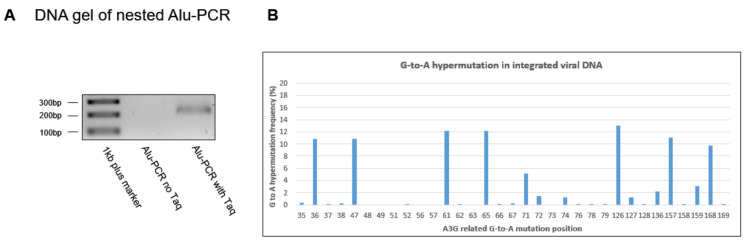
G-to-A hypermutation rate in integrated cDNA. Viral cDNA isolated from cells 19 h post-IIIB/H9 infection (from Figure 1) was subject to nested Alu PCR. The input cDNA concentration was adjusted to ensure that only samples with PCR Taq DNA polymerase in the first round of PCR reaction showed PCR product (**A**). The Alu PCR product was analyzed using the NGS-based G-to-A hypermutation-detecting assay (**B**). The results shown are representative of two independent experiments.

Our data showed that un-impacted viral gRNA was preferentially selected for viral RNA packing. It would be interesting to test whether the viral DNA integration step plays a role in the selection process. DNA samples from Figure 1A were subjected to a nef-Alu nested PCR assay to analyze the integrated proviral cDNA. To ensure the PCR product was integrated DNA-related, we adjusted our input DNA to make sure that the PCR product only showed up in the samples with Taq DNA polymerase added in the nef-Alu PCR (Figure 5A). The nested nef-Alu PCR product was subjected to G-to-A hypermutation analysis using the Genewiz Amplicon EZ service. The result showed no significant change in G-to-A hypermutation frequency in viral integrated DNA (Figure 5A) compared to viral cDNA (Figure 1A), suggesting that the selection of un-impacted gRNA preferentially encapsidated into the virion is after viral DNA integration. 

## 4. Discussion

The encapsidation of A3G into the HIV-1 particle is crucial for its antiviral activity against HIV-1 replication [1,2,3]. A3G is encapsidated into progeny virions and exerts its potent antiviral effects upon infection in a new cell by reducing cDNA production, inducting G-to-A hypermutations in the viral genome through its cytidine deaminase activity and causing defects in integration into the host genome. HIV-1 encodes Vif to counteract the antiviral function of A3G by excluding it from encapsidation and other mechanisms. However, evidence showed that detectable A3G cytidine deaminase activity remains in wild-type HIV-1 virion. In this study, we sought to study the role of encapsidated A3G in wild-type HIV-1 replication. We show that upon infection in Sup-T1 cells, encapsidated A3G from IIIB/H9 and NL4-3/A3G viruses induced G-to-A hypermutation in the viral cDNA. IIIB/Sup-T1 and NL4-3 failed to induce G-to-A hypermutation as expected due to the lack of endogenous A3G proteins in their producer cells (Sup-T1 and 293T cells) (Figure 1). Furthermore, our highly sensitive NGS-based G-to-A hypermutation assay showed that no significant G-to-A hypermutation frequency increase was observed in newly released viruses even though significant G-to-A hypermutation was detected in integrated viral DNA. 

**Figure 6:**
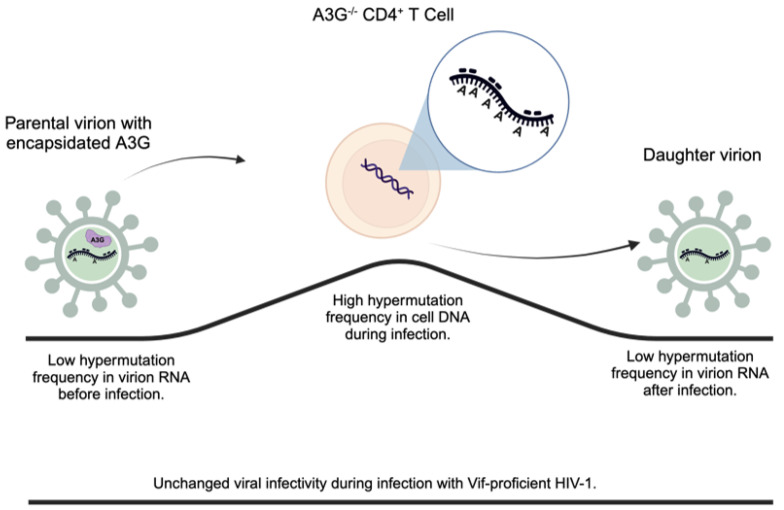
Working model. The parental virion containing encapsidated A3G initially exhibits a low hypermutation frequency prior to infecting A3G^−/−^ CD4^+^ T cells. However, during infection, integrated viral DNA undergoes significant G-to-A hypermutation. Interestingly, the daughter virion demonstrates a selective incorporation of RNA with a low G-to-A hypermutation frequency. Despite the increase of G-to-A hypermutation in viral cDNA, the viral infectivity remains unchanged.

This data are consistent with a previous study, reported by Rebecca et al., that showed evidence of a novel G-to-A hypermutation gradient that exists in the viral DNA, cellular RNA, and viral RNA at high, intermediate, and low mutational frequencies using an HIV-1 Vif YRHHY>A5 mutant virus [42]. Together with our findings using wild-type HIV-1 virus, we conclude that even though higher G-to-A hypermutation frequencies were detected in integrated viral DNA, lower G-to-A hypermutation frequencies were found in the viral RNA genome regardless of HIV-1 Vif function, as depicted in Figure 6. 

Even though substantial G-to-A hypermutation was found in integrated viral DNA, viral infectivity was not impacted. It showed a disconnection between G-to-A hypermutation in viral cDNA and viral infectivity. However, when we compare the G-to-A hypermutation frequency between the input virus for the infection and the newly released virus, we found the G-to-A hypermutation frequency was basically unchanged in the viral RNA genome of the two viruses (Figure 4). Traditionally, G-to-A hypermutation in viral cDNA has been considered a hallmark of A3G antiviral function, and measuring G-to-A hypermutation in viral cDNA has been routinely used to determine A3G antiviral activity. Our data suggest that measuring the G-to-A hypermutation of viral genome RNA, instead of viral cDNA, should be considered for determining A3G antiviral activity. 

It has been proposed that there is a delicate balance between A3G and Vif [1]. Vif uses multiple strategies to control A3G for its maximum benefits, including excluding A3G from progeny virions through A3G degradation and other mechanisms [2], directly inhibiting A3G cytidine deaminase activity [43,44,45], and reducing viral cDNA G-to-A hypermutation [46], etc. The lower frequency of hypermutation in the viral RNA genome suggests a selection mechanism for un-impacted viral RNA to be preferentially packaged into viral particles, as shown by us and Russell et al. [42]. The novel mechanism, possibly during integrated viral DNA transcription and/or viral genome RNA packaging into progeny virions, is utilized by HIV-1 to overcome the effects of A3G during infection. Interestingly, studies showed that the packaging of HIV-1 viral genomic RNA is heavily influenced by the “GGG tract” downstream of the TATA-box in the 5′ LTR. The G1-form transcript, transcribed from the third deoxyguanosine of the GGG tract, was preferred for incorporation into progeny virions [47,48,49]. As GG is the preferential target for A3G cytidine deaminase to convert GG to GA, A3G may disrupt the signature of G1-from transcript by mutating the first G-to-A, rendering it unfavorable for RNA packaging. Interestingly, a recent study showed that HIV-1 with a mutation at G3 or G2G3 exhibited replication fitness defects [50], a phenotype considered closely related to A3G function. Future systematic studies are warranted to reveal more details of the influence of A3G on viral genomic RNA packaging and replication fitness.

## Data Availability

The original contributions presented in the study are included in the article. Further inquiries can be directed to the corresponding author.

## Supplementary Materials

The following supporting information can be downloaded at https://www.mdpi.com/article/10.3390/v16050728/s1: Figure S1: A 199 bp DNA fragment within the nef-LTR region of the HIV-1 IIIB genome was amplified by PCR and submitted for NGS-based G-to-A hypermutation analysis.
