# Supplementary material for: The Disassociation of A3G-Related HIV-1 cDNA G-to-A Hypermutation to Viral Infectivity
Source: Viruses. 2024 May 4;16(5):728. doi: 10.3390/v16050728 (PMC11126051; doi:10.3390/v16050728)
Supplement: Supplementary file 1 [file viruses-16-00728-s001.zip › viruses-2939018-supplementary.pptx]

## Slide 1
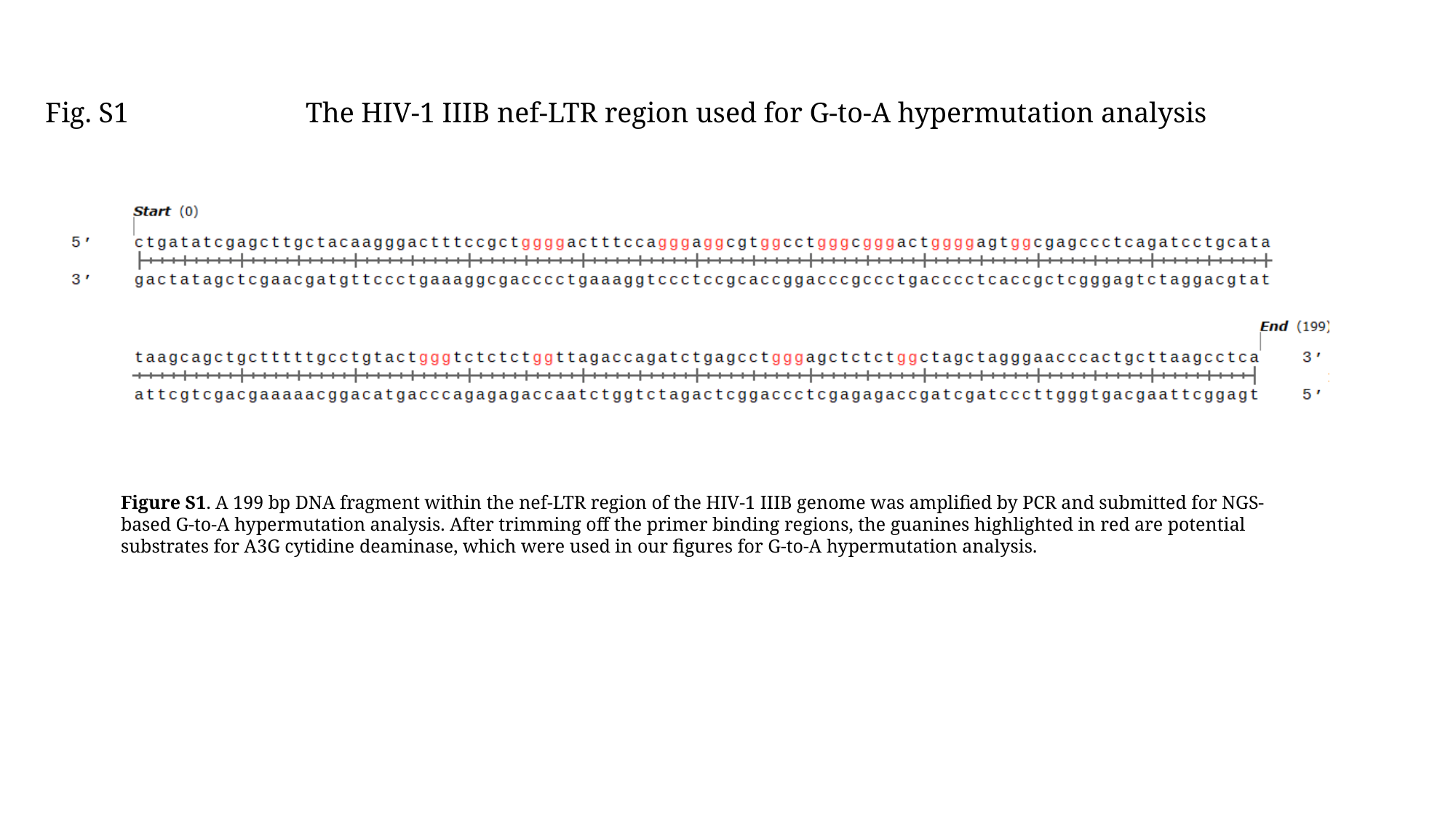

Fig. S1 The HIV-1 IIIB nef-LTR region used for G-to-A hypermutation analysis
Figure S1. A 199 bp DNA fragment within the nef-LTR region of the HIV-1 IIIB genome was amplified by PCR and submitted for NGS-based G-to-A hypermutation analysis. After trimming off the primer binding regions, the guanines highlighted in red are potential substrates for A3G cytidine deaminase, which were used in our figures for G-to-A hypermutation analysis.
